# Robust, microfabricated culture devices with improved control over the soluble microenvironment for the culture of embryonic stem cells

**DOI:** 10.1002/biot.201300245

**Published:** 2014-03-27

**Authors:** Rhys J Macown, Farlan S Veraitch, Nicolas Szita

**Affiliations:** Department of Biochemical Engineering, University College London London, UK

**Keywords:** Cell culture, Microfluidic culture device, Microreactor, Process development, Stem cells

## Abstract

The commercial use of stem cells continues to be constrained by the difficulty and high cost of developing efficient and reliable production protocols. The use of microfabricated systems combines good control over the cellular microenvironment with reduced use of resources in process optimization. Our previously reported microfabricated culture device was shown to be suitable for the culture of embryonic stem cells but required improvements to robustness, ease of use, and dissolved gas control. In this report, we describe a number of improvements to the design of the microfabricated system to significantly improve the control over shear stress and soluble factors, particularly dissolved oxygen. These control improvements are investigated by finite element modeling. Design improvements also make the system easier to use and improve the robustness. The culture device could be applied to the optimization of pluripotent stem cell growth and differentiation, as well as the development of monitoring and control strategies and improved culture systems at various scales.

## 1 Introduction

The commercial use of stem cells continues to be constrained by the difficulty and high cost of developing efficient and reliable production protocols [1]. The potential of stem cells has been widely reported; both for use in cell therapy applications and for the screening of drug candidates [2–4]. However, the number of factors that affect stem cell growth and differentiation, combined with their time-dependent optima, creates a wide experimental space to investigate [5, 6]. This issue is compounded by the high cost of many required medium components, limiting process optimization. As a result, many protocols for cell production remain largely non-optimized and poorly understood [7–9]. Consequently, cell yields can be very low and quality by design essentially impossible.

To address this challenge, we previously presented a microfabricated culture device for stem cell process development [10]. The reactor incorporated a traditional tissue-culture polystyrene (TC-PS) substrate as a growth surface. It also facilitated static cell seeding via a re-sealable lid. These features aimed to replicate traditional methods at a microfluidic scale. For increased control of the soluble environment, the reactor incorporated a closed culture chamber, of defined height, and perfusion of culture medium via a syringe pump. Finally, we demonstrated the quantitative monitoring of culture growth by phase contrast microscopy with novel image processing. This showed the system to be suitable for the co-culture of human embryonic stem cells on inactivated mouse embryonic fibroblasts.

Yet, this previously reported system required further improvements in two key areas. Firstly, the previous design had insufficient control over dissolved gas distribution. In addition to affecting growth rate, dissolved oxygen (DO) is also a key variable in various differentiation processes [11, 12]. However, previously the only source of oxygen was that arriving dissolved in medium at the inlet. This oxygen would be consumed at changing rates as medium flowed across the culture surface resulting in inconsistent and non-uniform concentrations. Secondly, the system needed to be made more robust and easier to use if it was to be widely adopted for use in process development [13, 14]. The previous system required the tightening of 10 screws to precise torques in order to achieve leak free operation. Further, a back pressure was required to prevent bubble formation in the culture chamber but a moderate increase in back pressure was liable to cause leaking.

In this report, we describe and model improvements in design intended to make a more controlled, more robust, and easier to use culture device. Changes to the layout of the fluidic channels improve the uniformity of medium flow across the chip, while alternate lid designs allow for greater control of dissolved gas concentrations in the culture chamber. New reactor packaging was designed to house three reactors. The redesigned packaging is easier to use and improves the burst pressure of the device by a factor of 6. This increased robustness enables improvement of a previously reported perfusion system [15]; introducing a backpressure regulator to prevent bubble formation. Preliminary culture results support the continued suitability of the system for the culture of adherent stem cells.

## 2 Materials and methods

### 2.1 Reactor design and construction

All custom parts were designed using SolidWorks® (SolidWorks, Cambridge, UK). The aluminum holder of the new design was made of anodized aluminum and manufactured by Finetech Engineering Limited (Hatfield, UK) to drawings produced from SolidWorks®. All other aluminum (3 mm, ALU-35, HABA, Cham, Switzerland and 6 mm, 188–264, RS, UK) and polycarbonate parts (3 mm, 681-637, RS, UK, and 5 mm, 681-659, RS, UK), including the interconnects, lids, top and bottom frames, and moulds, were manufactured by micro-milling as previously described [10]. Gaskets, gas-permeable lids, and the upper sections of the microfluidic chips were cast from poly(dimethylsiloxane) (PDMS) (Sylgard 184, Dow Corning, USA) in milled moulds and cured for 2 h at 90°C. The bottom layer of the microfluidic chip was produced by spin coating (P6708D, Specialty Coating Systems, USA) PDMS on a silanized (85041C, Sigma-Aldrich, UK) 4″ silicon wafer (Prolog Semicor, Ukraine) and curing at 90°C for 1 h. To bond PDMS parts, they were rinsed with ethanol, dried, and bonded using an air plasma (90 s, 30 W, 500 mTorr, PDC-002, Harrick Plasma, USA), and cured in an oven at 90°C for 2 h. The microscope slides used were made of TC-PS (16004, Nunc, Denmark).

### 2.2 Perfusion setup

A gas supply controlled by an electronic regulator (ITV0011-2BL, PNEU-STORE UK, Larkhall, UK) was connected via a 0.22 μm syringe filter and standard Upchurch® connectors to a length of 0.001″ internal diameter (ID) polyether ether ketone (PEEK) tubing submerged in a SCHOTT bottle of medium (1094367, 554-3004, 554-3000, 554-3005, VWR, Lutterworth, UK). Medium exited via a 70 cm length of 0.001″ ID PEEK tubing before being split to flow down each of three 50 cm lengths of 0.004″ ID PEEK tubing to the three parallel culture devices. Medium exiting each culture device passed through a 10 cm length of 0.03″ ID PEEK tubing before combining to flow through a 5 psi back pressure regulator (P-790, VWR) to a waste bottle. The flow rate at the outlet was monitored by a micro flow sensor (SLG1430-480, Sensirion, Staefa, Switzerland).

### 2.3 Finite element modeling

The flow of medium through different chip designs was analyzed by finite element analysis using COMSOL Multiphysics® 4.3b (COMSOL, Cambridge, UK). The fluidic networks were built as 3D models using SolidWorks® and imported as the geometry for the COMSOL® models. The fluid material was defined as water at 310.15 K but with the dynamic viscosity adjusted to 7.8 × 10^–4^ Pa s to more accurately represent culture medium [16]. A fully developed steady-state flow with no slip condition at the boundaries was assumed. The boundary conditions were set at the inlet to an average velocity calculated from the flow rate (300 μL h^–1^), and at the outlet to zero pressure. The geometry was divided into a free tetrahedral mesh with maximum and minimum element sizes of 40.6 and 0.05 μm, respectively resulting in 14 519 008 elements for the most complex chip design investigated. The models were solved using COMSOL's generalized minimal residual method (GMRES) iterative solver.

The distribution of DO was modeled in COMSOL by adding a transport of dilute species model to the existing fluid flow model. Cellular oxygen consumption was modeled as a constant flux through the culture surface of 5.6 × 10^–8^ mol m^–2^ s^–1^ reflecting a relatively high confluency of mouse embryonic fibroblasts between 1.9 × 10^5^ and 5.6 × 10^5^ cells cm^–2^ [17]. All other boundaries were set to zero flux. The diffusivity and concentration of DO in incoming medium were set to 2 × 10^–9^ m^2^ s^–1^ and 0.228 mM reflecting the diffusivity and solubility in medium in equilibrium with air at 1.2 atm and 298.15 K [18]. 1.2 atm is the approximate pressure at the inlet to the culture device. To model the gas-permeable lid, a 4.12 mm layer of PDMS was added above the culture chamber with a swept mesh and a diffusivity of 3.02 × 10^–8^ m^2^ s^–1^; the diffusivity in PDMS adjusted by the ratio of solubility in PDMS to solubility in medium [18, 19]. The upper surface of the PDMS layer was given a constant defined DO of 0.228 mM; the solubility in medium in equilibrium with air at 1.2 atm and 298.15 K [18]. The model was solved with a segregated solver with both segregations using COMSOL's GMRES iterative solver.

### 2.4 Static dissolved oxygen modeling

The steady-state pericellular DO concentration was calculated for both, cells under a 3 mm layer of medium, and cells in our device with a gas-permeable lid but no medium flow. The values for solubility and diffusivity used were as for finite element modeling. Fick's first law (Eq. 1) was used to solve for the equilibrium pericellular DO at oxygen consumptions ranging from 2.8 × 10^–10^ to 9.0 × 10^–9^ mol m^–2^ s^–1^ (covering the range expected to occur in monolayer mouse embryonic stem cell culture [17]).


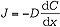
(1)

where *J* is the diffusion flux, *D* is the diffusivity, *C* is the concentration, and *x* the position in the direction of diffusion.

### 2.5 Frame bending calculations

A number of assumptions were made in order to approximate the bending of rigid frames. Firstly, the problem was treated as a bending beam problem rather than a bending plate problem. This simplification was made to allow simpler and more rapid investigation of potential bending effects. Secondly, the bending was simplified to a 2D problem by assuming no bending in the *xy* and *yz* planes (the *y* axis being parallel to a line from the inlet to the outlet and the *z*-axis normal to the culture surface). In the previously reported design, the screw forces were distributed evenly along the length of the chip so bending in the *yz* plane was likely to be negligible. With the new design, the clamping points of the aluminum holder were fewer and distributed non-symmetrically relative to the PDMS chip. However, bending in the *yz* plane was still likely to be negligible as the relevant second moments of inertia of the aluminum parts would be high relative to those relevant to bending in the *xz* plane. As there are no forces applied in the *x* and *y* directions, bending in the *xy* plane would not be expected. Finally, when calculating second moments of inertia, cross-sections were simplified to be symmetrical and some smaller features were ignored (Supporting information, C). This simplification to symmetry was necessary as symmetry is a key assumption of the calculation method used. For the previous design, the cross-sections were already symmetrical but for the new design, the recesses were shifted off center. Because height of cross-section is much more significant than breadth (Eq. 2), the effects of the simplification should be low. The smaller features that were ignored were small holes whose cross-section would have little impact on the second moment of inertia.

The second moments of inertia for each cross-section were calculated using the formula for a rectangle (Eq. 2) and parallel axis theorem (Eq. 3)


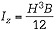
(2)

where *H* is the height (*z* dimension) and *B* is the breadth (*y* dimension).


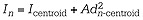
(3)

where *A* is the cross-sectional area and d is the distance between the center and the neutral axis.

The conjugate beam method was then applied to calculate the angle and displacement of the top and bottom frames at 50 μm intervals across the frame. The previous frame design (Supporting information, A) was modeled as beams supported by two pivots at the edges of the slide. One section of the new frame design (Supporting information, B) was modeled as beams fixed at both ends. The elastic modulus used for aluminum, polycarbonate, TC-PS, and PDMS were 7 × 10^10^, 2.3 × 10^9^, 3.0 × 10^9^, and 1.82 × 10^6^ Pa [20], respectively. The resultant force from compression of the PDMS chip was integrated numerically using the rectangle rule with a step distance of 50 μm. The axial force delivered by a screw was calculated using Eq. (4).


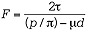
(4)

where *F* is the axial force, *τ* is the torque, *p* is the thread pitch (5 × 10^–4^ m), *μ* is the friction factor (assumed 0.5), and *d* is the pitch diameter (2.675 × 10^–3^ m).

### 2.6 Pressure measurements

To measure the pressure required to achieve a given flow, a 10 mL glass syringe (81660, Essex Scientific Laboratory Supplies, Benfleet, UK) was connected to one or more components via a 3-way valve (98-2750, Harvard Apparatus, UK). The third port of the 3-way valve was connected to a pressure sensor (40PC100G, Honeywell, USA) glued into an Upchurch® fitting with epoxy glue. A syringe drive was used to pump water at fixed flow rates and the pressure was logged via a LabVIEW™ routine (LabVIEW 2011, National Instruments, USA) and data acquisition card (USB-6009, National Instruments, USA).

To measure the burst pressure of a single reactor, the outlet was blocked with a 1/4-28 plug fitting and air was pumped at 7 mL min^–1^ using the above setup. The burst pressure was taken as the highest recorded applied pressure for a given experiment. The burst pressure was recorded three times for culture devices in each section of the new design. All device components used for burst pressure experiments had previously been autoclaved.

### 2.7 Cell culture

All parts of the new design were autoclaved along with all components of the perfusion system. The new culture system was then assembled in a sterile manner in a biological safety cabinet using sterile TC-PS slides (16004, Nunc). Two culture devices used the new chip design with perfusion barriers while the third used the previous chip design. The culture surfaces of the devices were coated with 0.1% gelatin (G1890, Sigma–Aldrich) for 15 min at room temperature. After removal of the gelatine, a suspension of E14 mouse embryonic stem cells (in culture medium) were seeded by pipette at a density of 7.3 × 10^4^ cells cm^–2^. Cells were allowed to settle and attach for 6 h before changing the medium. Culture medium consisted of knock-out Dulbecco's modified Eagle medium (10829-018) supplemented with 15% v/v fetal bovine serum (26140-079), Glutamax (35050-038), non-essential amino acids (11140-035), β-mercaptoethanol (31350-010, Life Technologies, UK), and leukemia inhibitory factor (130-095-779, Miltenyi Biotech, UK). The resealable lids were then inserted and screwed down. The polycarbonate section of the the lid for the chip of the previous design and one of the chips of the new design were solid while the second new chip used an open polycarbonate element.

The perfusion system was primed with medium and the medium reservoir filled with 200 mL of medium before connecting the tubing to the culture devices. The entire system was placed in a cage incubator (Okolab, Italy) heated to 37°C with the culture devices on the stage of an automated microscope (Eclipse Ti-E, Nikon Instruments, UK). Medium was perfused by a 20% O_2_ 5% CO_2_ gas mix (BOC, London, UK) for 84 h with phase contrast images regularly taken (DS-Fi1, Nikon Instruments). The pressure was initially adjusted to achieve a flow rate of 900 μL h^–1^ (300 μL h^–1^ in each chip) but was adjusted daily to compensate for changes in flow rate over time.

## 3 Results

### 3.1 Culture device design

As previously reported [10], culture devices consisted of a PDMS microfluidic chip compressed against a TC-PS microscope slide by a rigid frame (Supporting information, A). The PDMS chip consists of two layers, the upper containing the flow channels for medium perfusion and the lower raising these channels 120 μm above the culture plane. The culture surface has dimensions of 4 mm by 13 mm and is directly accessible by a re-sealable lid. When in place the lid defines the height of the culture chamber as 450 μm. The lid is sealed by compression of a PDMS gasket. The previous design compressed the chip and slide between top and bottom frames milled from 3 to 5 mm polycarbonate, respectively. The frames were clamped together with 10 screws tightened to 2 N cm. 5 mm thick polycarbonate interconnects with 1/4-28 fittings allowed connection with external fluidics. Lids were milled from 5 mm polycarbonate.

The design of the frame has been changed for this next generation of design. The bottom frame has been eliminated and the gasket chip and slide are now recessed into a 5 mm thick top frame. This whole assembly is then slid into one of three slots in an aluminum holder (Supporting information, B). The aluminum holder is assembled from four parts. The 5 mm thick top plate has three large windows where the interconnects and lid can be attached. The bottom plate milled from 8 mm aluminum contains three 5.55 mm deep cavities for the culture device assemblies. Three windows are positioned below the culture chambers and filled with a section of 2 mm polycarbonate, which protrudes 125 μm upward to provide additional compression around the edges of the culture chamber. Finally, two brackets attach to the top plate to support it on a microscope stage for imaging of cultures. Tightening the 12 screws attaching the top and bottom plates of the holder seals the three culture devices.

The durability of the interconnects has been improved by changing the material to 6 mm aluminum. The lid design has been changed to consist of a 4.1 mm gas permeable PDMS lid supported by a polycarbonate cap. The PDMS component defines the height of the culture chamber as 450 μm. The polycarbonate cap has three variations; one solid, one with a rectangular hole with the same cross-section as the culture surface, and one with a rectangular cavity with the same cross-section as the culture surface and holes to connect tubing for the continuous perfusion of a gas mixture.

### 3.2 Frame bending analysis

**Figure 1:**
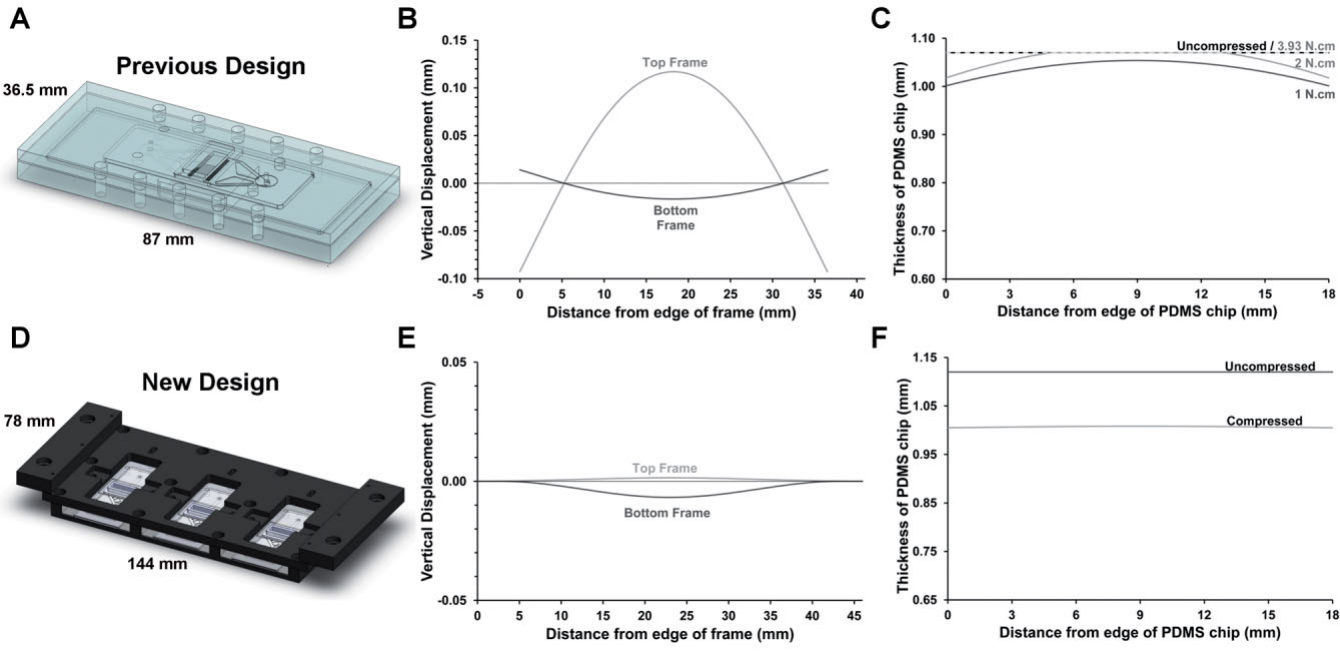
Approximations of frame bending of the previous frame design (A–C) and new frame design (D–F) from the conjugate beam method. The vertical displacement of the rigid components is shown (B, E) as well as the thickness of the PDMS chip with and without compression (C, F). For the previous design, the compression is shown at three different screw torques to demonstrate the sensitivity to clamping force. Note the different scales.

The approximate bending of the different rigid parts was calculated for both the previous and new frame designs (Fig. 1). The bending of the previous design was analyzed at three different screw torques to demonstrate the sensitivity to the axial force delivered. The maximum combined deflection of the previous design with 2 N cm of screw torque was 133 μm at the center of the chip. The maximum combined deflection of the new design was 8.2 μm.

### 3.3 Microfluidic chip

**Figure 2:**
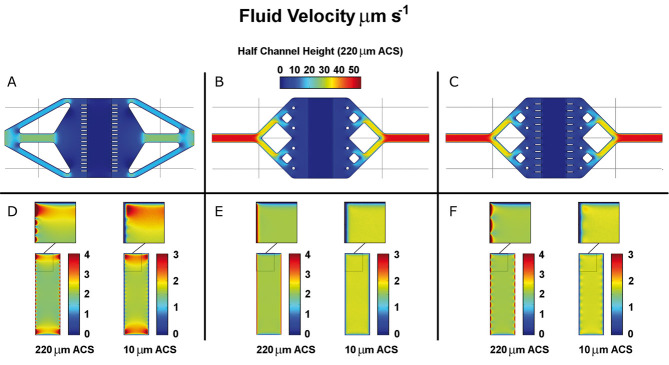
Results of COMSOL® finite element modeling of fluid flow in microfluidic chip designs. (A–C) The distribution of velocity magnitude at the center height of the channel, 220 μm above the culture surface (ACS) and (D–F) the distribution in the culture chamber at half channel height and 10 μm ACS. Sections of the culture chamber have been magnified in panels (D–F). Results are shown for the previous chip design (A, D), the new chip design without perfusion barriers (B, E), and the new chip design with perfusion barriers (C, F). All velocities are in μm s^–1^.

For the microfluidic chip, two iterations of design improvement were modeled by finite element analysis along with the previous design. The new designs use a tetrahedral tree-like structure to expand the flow [21]. One new design has no perfusion barriers bordering the culture chamber while the other uses arrays of 11 evenly space barriers on either side. Figure 2 shows the velocity distributions achieved with the three different designs at flow rates of 300 μL h^–1^. The mean shear stress 10 μm above the culture surface is 0.132 mPa in the old chip and 0.128 mPa in the two new chips. The distribution of shear stress has the same pattern as the distribution of velocity 10 μm above the culture plane.

### 3.4 Dissolved oxygen modeling

**Figure 3:**
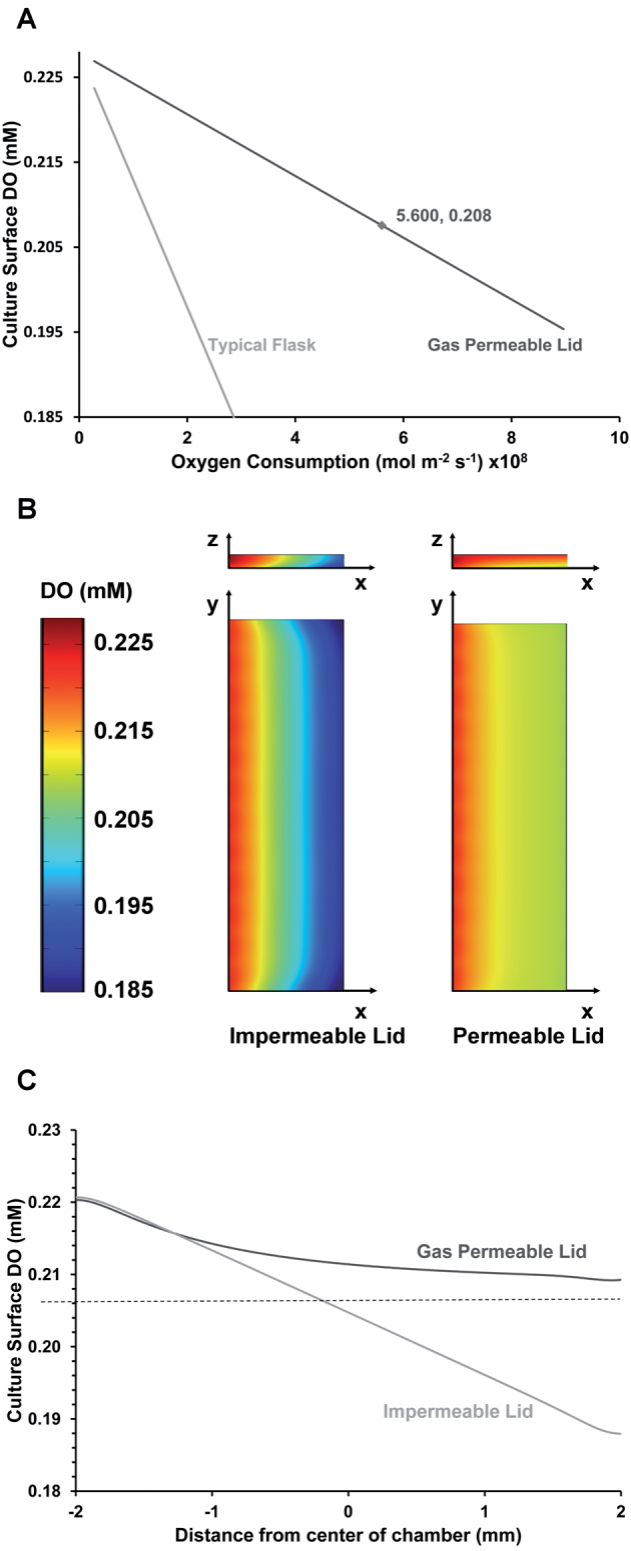
Modeling of the effect of a gas permeable lid on dissolved oxygen. (A) shows the equilibrium pericellular DO calculated using Fick's law for a static system. The culture device with a 4.1 mm gas permeable PDMS lid is compared to a flask or culture well with a 3 mm height of medium. (B) shows the distribution of DO at the culture surface and for a vertical slice along the center line, comparing culture devices with permeable and impermeable lids. (C) shows the variation in culture surface DO along the center line, comparing culture devices with permeable and impermeable lids. For panels (B) and (C), the culture devices are perfused at 300 μL h^–1^ with medium containing 0.228 mM of dissolved oxygen and cellular oxygen consumption was modeled as 5.6 × 10^–8^ mol m^–2^ s^–1^ reflecting a relatively high confluency of mouse embryonic fibroblasts.

The distribution of DO in the reactor was modeled with the new gas permeable lid design. Figure 3A shows how the pericellular DO of a static system changes with changing cellular oxygen uptake. A static culture device with a gas permeable lid is compared to a flask or culture well covered in 3 mm of medium (equivalent to ∼7.5 mL in a T-25 flask). Figure 3B and 3C shows the results of finite element modeling of culture devices with medium perfused at 300 μL h^–1^. Figure 3B compares the DO distribution with the new gas permeable lid and with the old impermeable lid. The DO distribution is shown both across the culture surface and for a vertical slice through the center of the chamber. Figure 3C shows how culture surface DO decreases along a line across the center of the culture surface. The mean culture surface DOs and their standard deviations are 0.204 ± 0.010 and 0.213 ± 0.003 mM for the gas impermeable and gas permeable lids, respectively.

### 3.5 Pressure measurements

The flow resistances of the three lengths of 0.004″ tubing were 1.55 × 10^14^, 1.57 × 10^14^, and 1.56 × 10^14^ Pa s m^–3^, respectively. The flow resistances of the three culture devices in the new design were 1.58 × 10^12^, 1.67 × 10^12^, and 1.14 × 10^12^ Pa s m^–3^, respectively. The third culture device used the previous microfluidic chip design. The range of culture device resistances is 37% of the mean value. However, the range of resistances for the culture device-tubing pairings is just 2% of the mean value. The expected backpressure at the inlet of a new device at a flow rate of 300 μL h^–1^ is 3.5 × 10^4^ Pa. The mean burst pressure of a single culture device in the new design is 3.7 × 10^5^ ± 1.2 × 10^5^ Pa (standard deviation *n* = 9) compared with 5.9 × 10^4^ ± 1.8 × 10^4^ Pa for the previous design [10]. At this point, loss of pressure occurs by reversible failure of a compression seal rather than permanent damage to a component. This is evidenced by repeatability of the burst pressure with the same parts.

### 3.6 Preliminary cell culture

**Figure 4:**
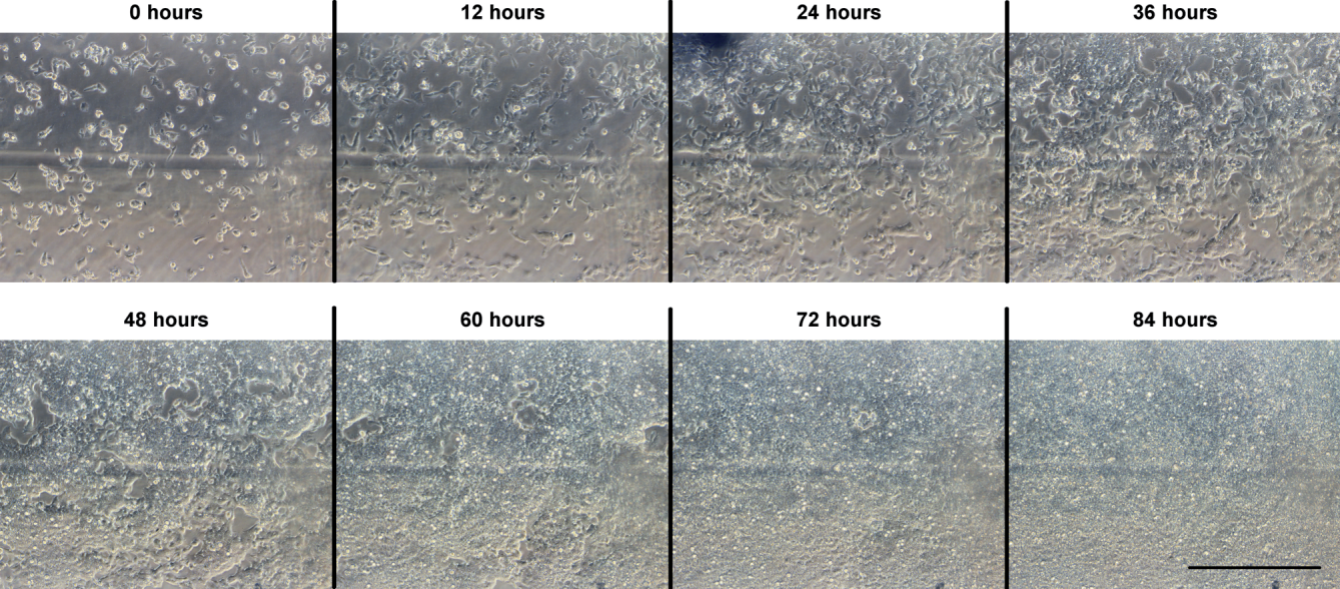
Phase contrast microscopy images of mouse embryonic stem cells in a culture device using the new chip design and a gas permeable lid. Images were taken in the same position at regular time intervals from the start of medium perfusion (0 h). Images were taken using a 10× objective and the scale bar represents 500 μm.

The mouse embryonic stem cells seeded by pipette settled and attached over 6 h before expanding in all three culture devices, under perfusion conditions. The cells reached over-confluence before the end of the culture. Figure 4 shows time course images of cells during perfusion culture in a device using the new chip design with a gas permeable lid.

## 4 Discussion

Improvements to the frame and interconnects make the described cultured device significantly more robust than the previous design. The reduced bending of the frame (Fig. 1B and 1E) results in a larger and more uniform compression of the PDMS chip (Fig. 1C and 1F). This in turn results in a significantly higher burst pressure of 3.7 × 10^5^ ± 1.2 × 10^5^ Pa, a factor of 6.3 higher than reported for the previous design [10]. Furthermore, unlike with the previous design, achieving this burst pressure does not require the precise application of screw torque. As seen in Fig. 1C, small differences axial force (delivered by screw torque) have a significant impact on the compression of the PDMS chip. Such variations are relatively common and can result from material and thread variations as well as imprecision in the applied torque. This increased robustness and simplicity of assembly is vital if the culture device is to be widely adopted for process development.

A more uniform compression will also contribute to achieving a more uniform velocity profile across the culture chamber by maintaining a uniform channel height. Achieving a uniform flow profile is important as it results in both the shear stress and soluble factor concentrations being more uniform across the culture surface. Thus, the conditions cells experience can be kept in a narrower band about the values being investigated. Two new chip designs were developed to improve flow uniformity. Both new designs used a tree like structure to expand flow more evenly; one of these designs also included perfusion barriers intended to improve uniformity by creating an array of equal, high-resistance flow paths. Fluid flow modeling of velocity profiles in uncompressed chips (Fig. 2) shows that both new chip designs deliver more uniform flow across the culture chamber than the previous chip design. Of the two new designs, the chip without perfusion barriers delivers the most uniform flow. The perfusion barriers included to improve uniformity actually reduce uniformity around the edges of the chip by creating small dead zones. However, in practice the flexible PDMS layers tend to contact and stick to each other without the support provided by the perfusion barriers making the barrier inclusive design preferred.

Further improvements are made to the control of the soluble environment by inclusion of gas permeable lids. As seen in Fig. 3A, the pericellular DO in a flask or culture well will drop dramatically as oxygen consumption increases with cell density. By contrast, even in static culture, the pericellular DO in a culture device with a gas permeable lid reduces far more gradually. The DO gradient could be reduced further if the thickness of the PDMS lid could be decreased. However, the gradient is more sensitive to the depth of the culture medium due to the lower solubility of oxygen in medium. At an oxygen consumption of 5.6 × 10^–8^ mol m^–2^ s^–1^, reflecting a relatively high density of mouse embryonic stem cells, COMSOL® modeling shows that the gas permeable lid increases the uniformity of pericellular DO (Fig. 3B and 3C). At the relatively high flow rate of 300 μm h^–1^ used in previous experiments, the uniformity of culture surface DO is significantly increased by use of a gas permeable lid. Uniformity could be further reduced at medium flow rates lower than the 300 μm h^–1^ modeled; due to the lower driving pressure required the partial pressure of oxygen at the inlet would be lower and the DO profile would flatten toward a constant value equivalent to the static equilibrium value of 0.208 mM (Fig. 3C).

For convenience, the new holder is designed to house three culture devices side by side and achieve compression seals with the tightening of just 12 screws (compared to the 30 previously required). Medium can be perfused through these three devices using a gas driven perfusion system as previously required. The increased burst pressure of the system allows the incorporation of a 5 psi backpressure regulator at the outlet. This backpressure is included to force out any un-dissolved gases via the permeable PDMS. Equal flow through the three devices is achieved by the inclusion of equal lengths of highly resistive tubing in each flow path. This system should therefore allow either experimentation in triplicate, for more statistically significant results, or the comparison of different culture device setups with cells from the same passage.

The system was used for a preliminary perfusion culture experiment using E14 mouse embryonic stem cells. After allowing cells to settle and attach for 6 h, medium was perfused for 84 h and cells were monitored by phase contrast microscopy. The perfusion system is yet to be optimized and changes in flow rate, observed at a constant inlet pressure, indicate feedback control may be required. However, cells in all three culture devices expanded over the course of perfusion culture, which was continued to over-confluence. Following culture experiments with the previous culture system [10], these preliminary results support the continued suitability of the system for the culture of adherent stem cells. In summary, the combined improvements described in this report significantly increase the level of control over a number of key process parameters while also making the culture device a more robust and powerful tool for stem cell process development.

## Abbreviations

DO: dissolved oxygen
GMRES: generalized minimal residual method
ID: internal diameter
PDMS: poly(dimethylsiloxane)
PEEK: polyether ether ketone
TC-PS: tissue-culture polystyrene

## Supporting Information

As a service to our authors and readers, this journal provides supporting information supplied by the authors. Such materials are peer reviewed and may be re-organized for online delivery, but are not copy-edited or typeset. Technical support issues arising from supporting information (other than missing files) should be addressed to the authors.

suppinfo

## References

[1] Sharkis SJ, Jones RJ, Civin C, Jang Y-Y (2012). Pluripotent stem cell-based cancer therapy: Promise and challenges. Sci. Transl. Med.

[2] Martínez-Morales PL, Revilla A, Ocaña I, González C (2013). Progress in stem cell therapy for major human neurological disorders. Stem Cell Rev. Rep.

[3] Migliaccio AR, Whitsett C, Papayannopoulou T, Sadelain M (2012). The potential of stem cells as an in vitro source of red blood cells for transfusion. Cell Stem Cell.

[4] Volarevic V, Ljujic B, Stojkovic P, Lukic A (2011). Human stem cell research and regenerative medicine – present and future. Br. Med. Bull.

[5] Gadjanski I, Spiller K, Vunjak-Novakovic G (2012). Time-dependent processes in stem cell-based tissue engineering of articular cartilage. Stem Cell Rev. Rep.

[6] Wagers AJ (2012). The stem cell niche in regenerative medicine. Cell Stem Cell.

[7] Serra M, Brito C, Sousa MFQ, Jensen J (2010). Improving expansion of pluripotent human embryonic stem cells in perfused bioreactors through oxygen control. J. Biotechnol.

[8] King JA, Miller WM (2007). Bioreactor development for stem cell expansion and controlled differentiation. Curr. Opin. Chem. Biol.

[9] Kirouac DC, Zandstra PW (2008). The systematic production of cells for cell therapies. Cell Stem Cell.

[10] Reichen M, Macown RJ, Jaccard N, Super A (2012). Microfabricated modular scale-down device for regenerative medicine process development. PLoS ONE.

[11] Funamoto K, Zervantonakis IK, Liu Y, Ochs CJ (2012). A novel microfluidic platform for high-resolution imaging of a three-dimensional cell culture under a controlled hypoxic environment. Lab Chip.

[12] Fernandes TG, Diogo MM, Fernandes-Platzgummer A, da Silva CL, Cabral JMS (2010). Different stages of pluripotency determine distinct patterns of proliferation, metabolism, and lineage commitment of embryonic stem cells under hypoxia. Stem Cell Res.

[13] Skafte-Pedersen P, Sip CG, Folch A, Dufva M (2013). Modular microfluidic systems using reversibly attached PDMS fluid control modules. J. Micromech. Microeng.

[14] Tkachenko E, Gutierrez E, Ginsberg MH, Groisman A (2009). An easy to assemble microfluidic perfusion device with a magnetic clamp. Lab Chip.

[15] Reichen M, Veraitch FS, Szita N (2013). Development of a multiplexed microfluidic platform for the automated cultivation of embryonic stem cells. J. Lab Autom.

[16] Bacabac RG, Smit TH, Cowin SC, Van Loon JJWA (2005). Dynamic shear stress in parallel-plate flow chambers. J. Biomech.

[17] Powers DE, Millman JR, Huang RB, Colton CK (2008). Effects of oxygen on mouse embryonic stem cell growth, phenotype retention, and cellular energetics. Biotechnol. Bioeng.

[18] Nishikawa M, Kojima N, Komori K, Yamamoto T (2008). Enhanced maintenance and functions of rat hepatocytes induced by combination of on-site oxygenation and coculture with fibroblasts. J. Biotechnol.

[19] Merkel TC, Bondar VI, Nagai K, Freeman BD, Pinnau I (2000). Gas sorption, diffusion, and permeation in poly(dimethylsiloxane). J. Polym. Sci. B: Polym. Phys.

[20] Schneider F, Fellner T, Wilde J, Wallrabe U (2008). Mechanical properties of silicones for MEMS. J. Micromech. Microeng.

[21] Saias L, Autebert J, Malaquin L, Viovy J-L (2011). Design, modeling and characterization of microfluidic architectures for high flow rate, small footprint microfluidic systems. Lab Chip.

