# Supplementary material for: Robust, microfabricated culture devices with improved control over the soluble microenvironment for the culture of embryonic stem cells
Source: Biotechnol J. 2014 Mar 27;9(6):805–13. doi: 10.1002/biot.201300245 (PMC4674967; doi:10.1002/biot.201300245)
Supplement: Supplementary file 1 — suppinfo [file biot0009-0805-sd1.pdf]

Biotechnology
Journal

Supporting Information for DOI 10.1002/biot.201300245

Robust, microfabricated culture devices with improved control over the soluble microenvironment for the culture of embryonic stem cells

Rhys J Macown, Farlan S. Veraitch, Nicolas Szita

© 2014 Wiley-VCH Verlag GmbH & Co. KGaA, Weinheim

Supporting Information

Supporting information A shows exploded and assembled views of the previous culture device design.

Supporting information B shows assembled and exploded views of the new culture system design.

Supporting information C shows the calculation of second moments of inertia used in the frame bending calculations. It includes schematics of the simplified cross sections used. Below each section are calculations of the second moments of inertia and elastic moduli for the different parts of that cross section.

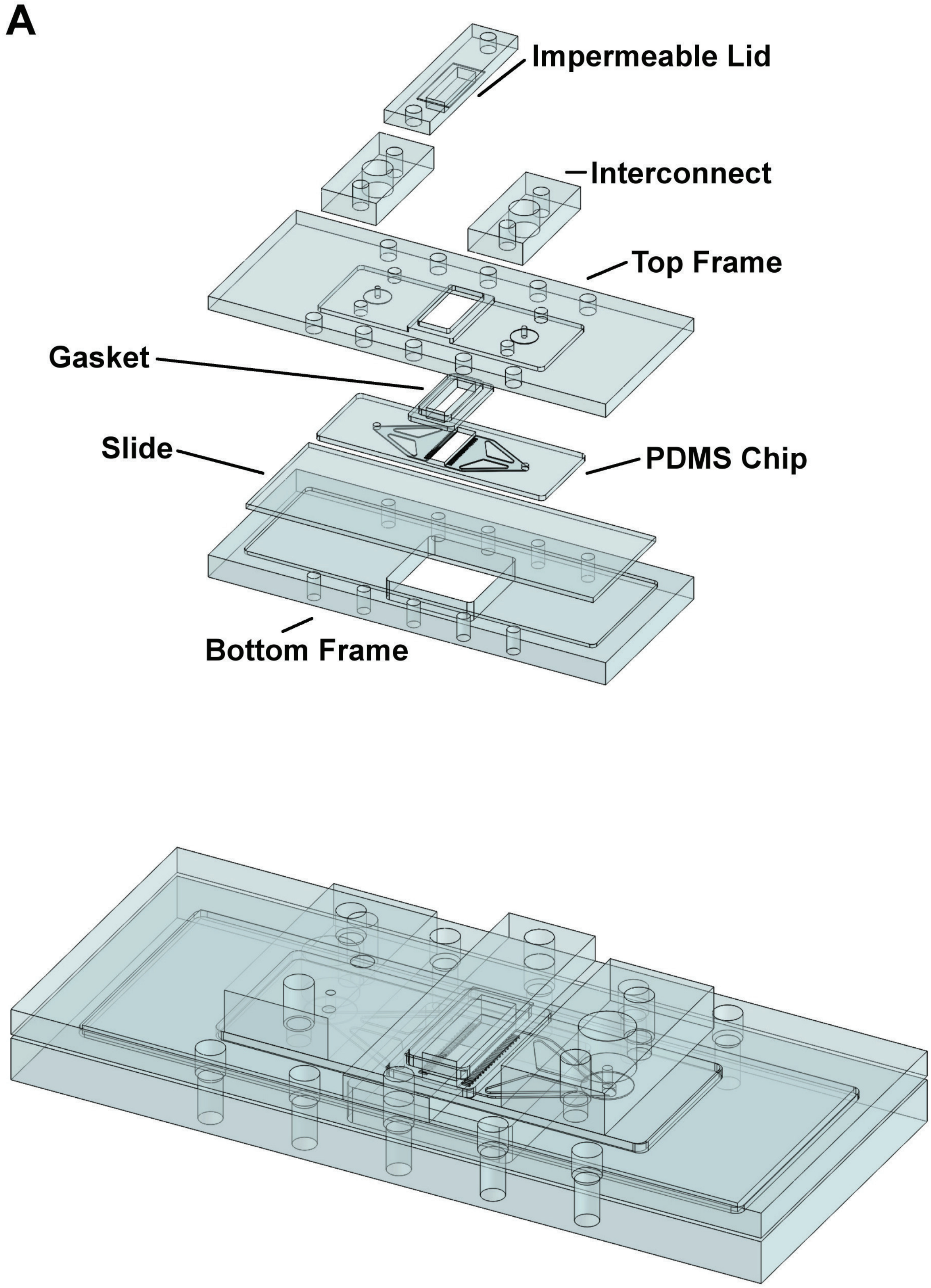
A
Impermeable Lid
Interconnect
Top Frame
Gasket
Slide
PDMS Chip
Bottom Frame

B

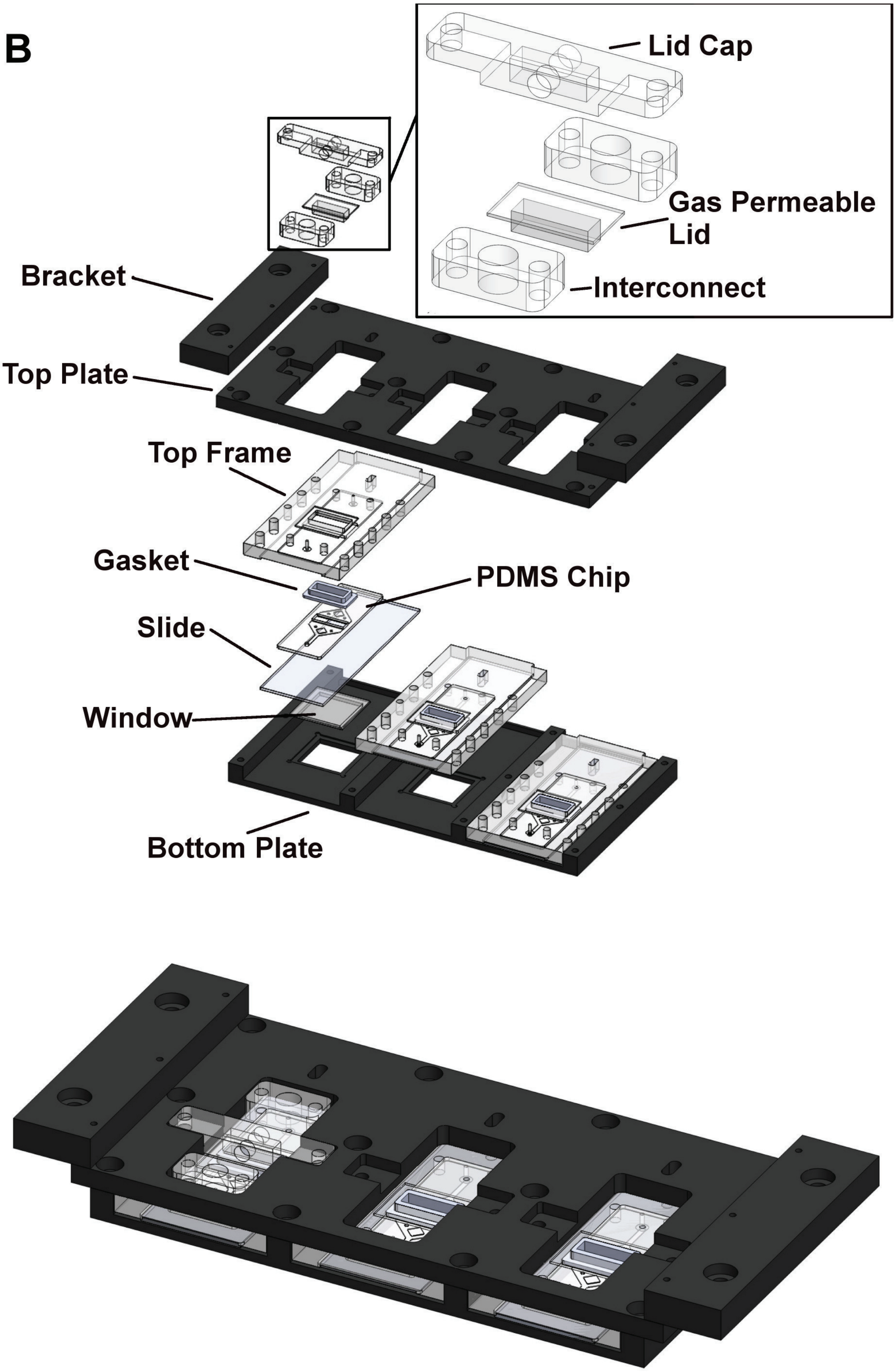
Lid Cap
Gas Permeable Lid
Interconnect
Bracket
Top Plate
Top Frame
Gasket
PDMS Chip
Slide
Window
Bottom Plate

C Previous Design - Top Frame

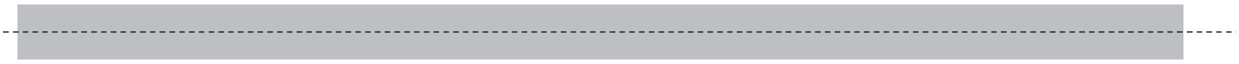

Top frame section 1 - 3 mm solid polycarbonate

B	8.70E-02	m
H	3.00E-03	m
I	1.96E-10	m^4
E - Polycarbonate	2.30E+09	Pa
EI	4.50E-01	$Pa.m^4$

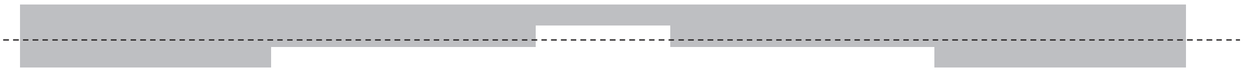

Top frame section 2 - Chip and Gasket Recesses

Chip recess b	4.95E-02	m	Gasket recess b	1.00E-02	m
Chip recess h	9.50E-04	m	Gasket recess h	1.00E-03	m
Chip recess I	3.54E-12	m^4	Gasket recess I	8.33E-13	m^4
Chip recess area	4.70E-05	m^2	Gasket recess area	1.00E-05	m^2
Chip axis distance	1.03E-03	m	Gasket axis distance	5.00E-05	m
adjusted Chip I	5.29E-11	m^4	adjusted Gasket I	8.58E-13	m^4
total I	1.41949E-10	m^4			
**EI**	**0.326483515**	**$Pa.m^4$**			

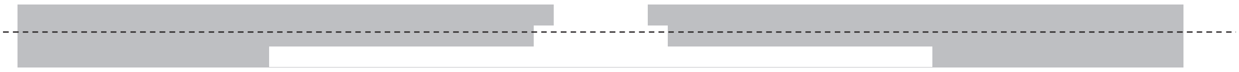

Top frame section 3 - opening for culture chamber axis

Opening b	7.00E-03	m
Opening h	1.05E-03	m
Openign recess I	6.75E-13	m^4
Opening recess area	7.35E-06	m^2
Opening axis distance	1.25E-05	m
adjusted Opening I	6.76E-13	m^4
total I	1.41E-10	m^4
**EI**	**3.25E-01**	**$Pa.m^4$**

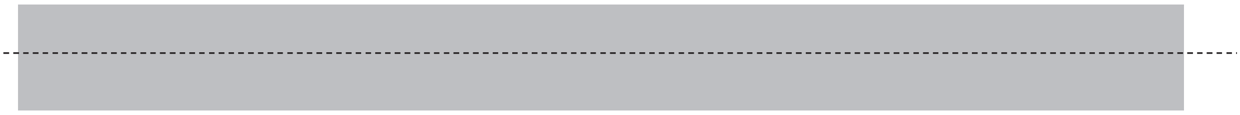

Bottom frame section 1- 5 mm solid polycarbonate

B	8.70E-02	m
H	5.00E-03	m
I	9.06E-10	m^4
E - Polycarbonate	2.30E+09	Pa
**EI**	**2.08E+00**	**$Pa.m^4$**

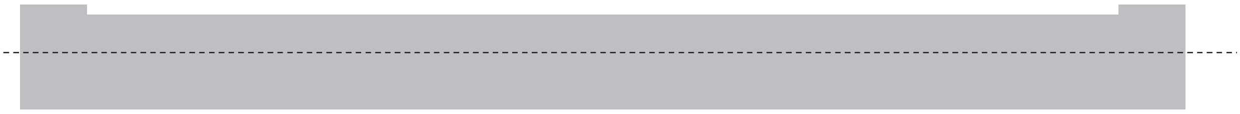

Bottom frame section 2 - slide recess

Slide recess b	7.70E-02	m
Slide recess h	7.50E-04	m
Slide recess I	2.71E-12	m^4
Slide recess area	5.78E-05	m^2
Slide recess axis distan	2.13E-03	m
adjusted Slide recess I	2.63E-10	m^4
total I	6.43E-10	m^4
**EI**	**1.48E+00**	**$Pa.m^4$**

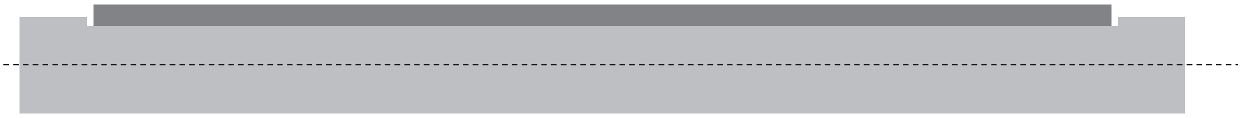

**Bottom frame section 3 - TC-PS Slide**		
Slide b	7.60E-02	m
Slide h	1.00E-03	m
Slide I	6.33E-12	m^4
Slide area	7.60E-05	m^2
Slide axis distance	2.25E-03	m
adjusted Slide I	3.91E-10	m^4
E - TCPS	3.00E+09	Pa
**Total EI**	**2.65E+00**	**Pa.m^4**

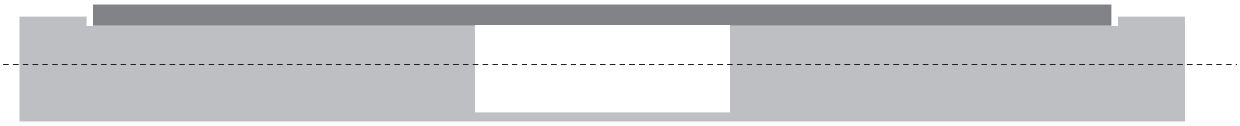

**Bottom frame section 4 - culture chamber viewing window**		
window b	1.90E-02	m
window h	4.25E-03	m
window recess I	1.22E-10	m^4
windowt recess area	8.08E-05	m^2
window axis distance	3.75E-04	m
adjusted window I	1.33E-10	m^4
E - PC	2.30E+09	Pa
**Total EI**	**2.35E+00**	**Pa.m^4**

New Design

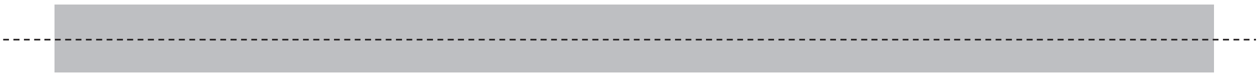

Top plate section 1 - 5 mm solid aluminium

B	7.80E-02	m
H	5.00E-03	m
I	8.13E-10	m^4
E - Aluminium	7.00E+10	Pa
**EI**	5.69E+01	$Pa.m^4$

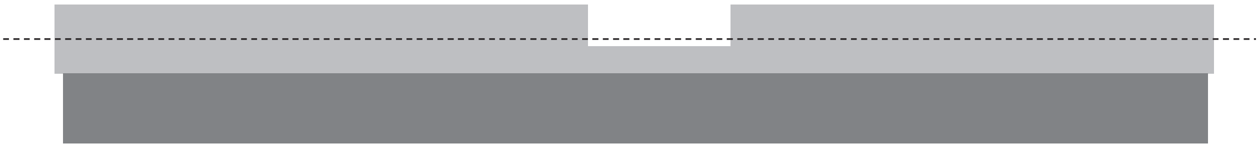

Top plate section 2 - Lid recess and 5 mm polycarbonate top plate

Recess b	1.00E-02	m	Top plate b	7.70E-02	m
Recess h	3.00E-03	m	Top plate h	5.00E-03	m
I	2.25E-11	m^4	I	8.02E-10	m^4
A	3.00E-05	m^2	A	3.85E-04	m^2
d	1.00E-03	m	d	5.00E-03	m
adjusted I	5.25E-11	m^4	adjusted I	1.04E-08	m^4
			E - Polycarbonate	2.30E+09	Pa
**EI**	**7.72E+01**	**$Pa.m^4$**			

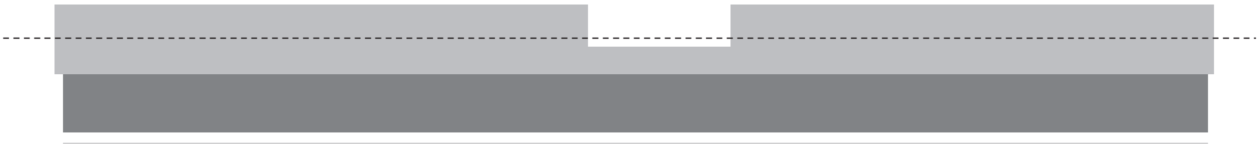

Top plate section 3 - Slide recess

Recess b	7.70E-02	m
Recess h	7.50E-04	m
I	2.71E-12	m^4
A	5.78E-05	m^2
d	7.13E-03	m
adjusted I	2.93E-09	m^4
**EI**	**7.04E+01**	**$Pa.m^4$**

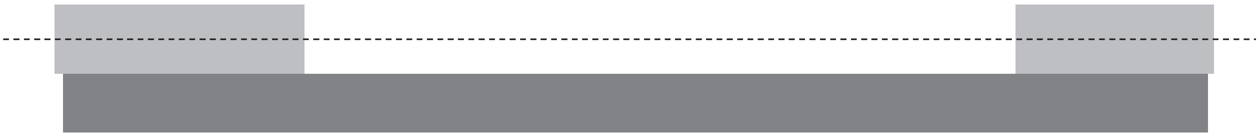

Top plate section 4 - Viewing window

Window b	5.00E-02	m
Window h	5.00E-03	m
I	5.21E-10	m^4
**EI**	**3.40E+01**	**$Pa.m^4$**

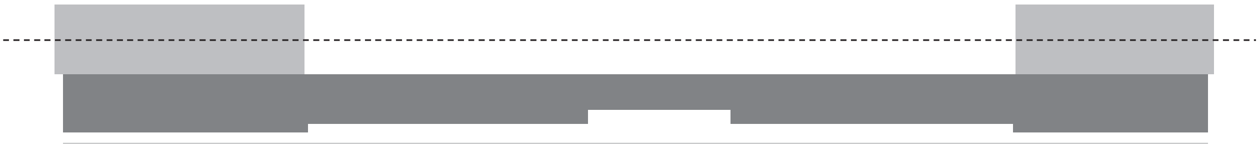

Top plate section 5 - slide and gasket recesses

chip recess b	4.95E-02	m	gasket recess b	1.00E-02	m
chip recess h	7.00E-04	m	gasket recess h	1.00E-03	m
I	1.41E-12	m^4	I	8.33E-13	m^4
A	3.47E-05	m^2	A	1.00E-05	m^2
d	6.40E-03	m	d	5.55E-03	m
adjusted I	1.42E-09	m^4	adjusted I	3.09E-10	m^4
**EI**	**3.00E+01**	**$Pa.m^4$**			

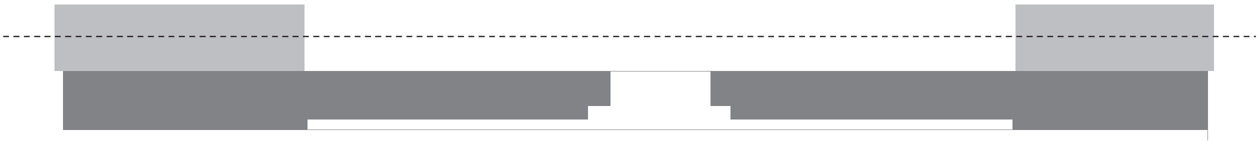

Top plate section 6 - opening for culture chamber access

opening b	7.00E-03	m
opening h	2.55E-03	m
I	9.67E-12	m^4
A	1.79E-05	m^2
d	3.78E-03	m
adjusted I	2.64E-10	m^4
**EI**	**2.94E+01**	**$Pa.m^4$**

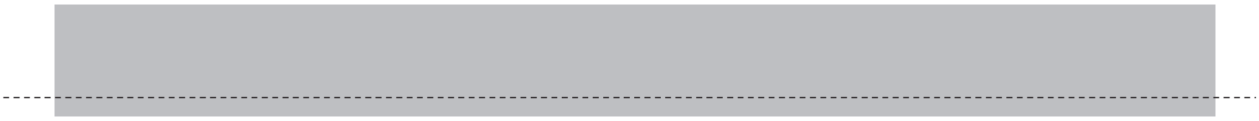

Bottom - section 1

B	7.80E-02	m
H	8.00E-03	m
I	3.33E-09	m^4
A	6.24E-04	m^2
d	2.78E-03	m
adjusted I	8.13E-09	m^4
E - Aluminium	7.00E+10	Pa
**EI**	**5.69E+02**	**$Pa.m^4$**

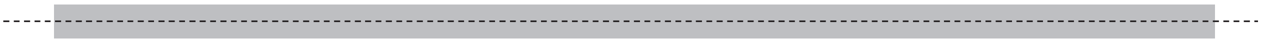

Bottom plate section 2 - culture device recess

remainin B	7.80E-02	m
remaining H	2.45E-03	m
I	9.56E-11	m^4
**EI**	**6.69E+00**	**$Pa.m^4$**

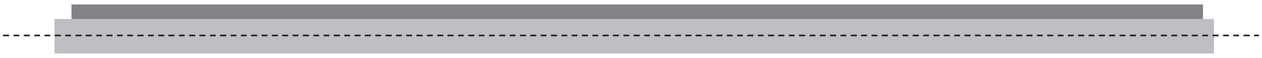

Bottom plate section 3 - slide

Slide B	7.60E-02	m
Slide H	1.00E-03	m
I	6.33E-12	m^4
A	7.60E-05	m^2
d	1.73E-03	m
adjusted I	2.32E-10	m^4
E - TCPS	3.00E+09	Pa
**EI**	**7.39E+00**	**$Pa.m^4$**

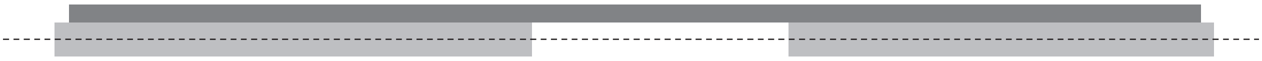

Bottom plate section 4 - culture chamber viewing window

Window B	1.80E-02	m
Window H	2.45E-03	m
I	2.21E-11	m^4
**EI**	**5.84E+00**	**$Pa.m^4$**
